# Value of multi-detector computed tomography combined with serum tumor markers in diagnosis, preoperative, and prognostic evaluation of pancreatic cancer

**DOI:** 10.1186/s12957-022-02785-x

**Published:** 2022-09-29

**Authors:** Jianli Su, Yunfeng Wang, Hua Shao, Xinting You, Shuying Li

**Affiliations:** 1 Department of Clinical Laboratory, Qilu Hospital (Qingdao), Cheeloo College of Medicine, Shandong University, Qingdao, 266035 China; 2 Department of Clinical Laboratory, Chengyang People’s Hospital, Qingdao, 266109 China; 3 Radiophysics Department, The Affiliated Qingdao Central Hospital of Qingdao University, The Second Affiliated Hospital of Medical College of Qingdao University, Qingdao, 266042 China; 4 Department of Endoscopic Diagnosis and Treatment, Qingdao Eighth People’s Hospital, Qingdao, 266100 China; 5 Department of Hepatobiliary Pancreatic Surgery (I), Central Hospital Affiliated to Shandong First Medical University, Jinan, 250013 China

**Keywords:** Pancreatic cancer, Multi-detector computed tomography, CA199, CA242, CEA

## Abstract

**Background:**

Multi-detector computed tomography (MDCT) and serum tumor markers are commonly used in the diagnosis of pancreatic cancer (PC). In this article, we focused on the evaluation of the clinical value of MDCT combined with serum tumor markers CA199, CA242, and CEA in diagnosis, preoperative, and prognostic evaluation of PC.

**Methods:**

Eighty-five PC patients (PC group) and 39 patients with pancreatitis (control group) admitted to our hospital were selected for our present research study. MDCT, CA199, CA242, and CEA examination were examined in all patients, and their value in diagnosis, preoperative, and prognostic evaluation of PC was retrospectively analyzed.

**Results:**

There were 69 patients whose clinical staging results of MDCT were consistent with the postoperative pathological diagnosis. The coincidence rate was 70.00% in stage I, 62.96% in stage II, 72.72% in stage III, and 80.00% in stage IV, respectively, and the overall coincidence rate was 69.57%The levels of CA199, CA242, and CEA in PC group were remarkably higher than those in control group and were sharply correlated with clinical stage, differentiation degree, and distant metastasis. The sensitivity, accuracy, and negative predictive value of MDCT combined with serum CA199, CA242 and CEA in the diagnosis of PC were significantly improved compared with those of each single test. In PC group, the 2-year event-free survival rate of the group with high CA199, CA242, and CEA expression was remarkably lower than that of the low expression group.

**Conclusion:**

MDCT combined with CA199, CA242, and CEA notably improved the diagnostic efficiency of PC and had guiding significance for preoperative and prognostic evaluation of PC.

## Introduction

Pancreatic cancer (PC) is a ductal adenocarcinoma mostly originating from the glandular epithelium, which is a common malignant tumor of the digestive system. Pancreatic cancer is one of the malignant tumors with a high degree of malignancy and a seriously poor prognosis [1]. With the increase of work pressure, the fast pace of life and the change of diet structure, the incidence of PC has been on the rise worldwide [2]. The pancreas is located behind the peritoneum of human body and is hidden, with many blood vessels and other organs distributed nearby, so it is different to detect PC early. Moreover, there are no typical clinical symptoms in the early stage and the diagnosis is often in the middle and late stages, leading to a poor prognosis [3]. It is reported that the 5-year survival rate of advanced PC is less than 5% [4]. Therefore, early diagnosis of PC, correct assessment of the disease before treatment and formulation of the best treatment are of great significance for improving the prognosis [5]. Imaging is an important means for the diagnosis of PC. Ultrasound is often used as the first choice for the diagnosis of PC, due to its low cost and non-invasive characteristics. However, due to the deep anatomical position of the pancreas and the influence of anterior intestinal gas, it is not effective in identifying small lesions, differentiating benign and malignant tumors and tumor invasion, and is not suitable for the early diagnosis of PC [6]. Multi-detector computed tomography (MDCT) is one of the most commonly used imaging techniques for the diagnosis of PC, which can provide an objective basis for the diagnosis and preoperative clinical staging evaluation [7]. Serum tumor markers are not only used in the early diagnosis, but also have certain value in prognostic evaluation as quantitative indicators in PC [8].

In this study, 85 patients with PC were retrospectively analyzed to investigate the clinical value of MDCT combined with serum markers, and to provide reference significance for the early diagnosis of PC.

## Materials and methods

### Clinical data

From January 2016 to December 2018, 85 patients with PC (PC group) and 39 patients with pancreatitis (control group) admitted to Qilu Hospital (Qingdao) were selected as the research objects. PC group: There were 46 cases of painless jaundice, 80 cases of upper abdominal discomfort, and 73 cases of weight loss. There were 56 males and 29 females. The age range was 35–69 years, with an average of 51.26 ± 8.94 years. Control group: there were 25 males and 14 females. The age ranged from 34 to 70 years, with an average of 52.10 ± 9.03 years. Inclusion criteria were as follows: ① patients were confirmed by histopathology, ② patients were newly diagnosed, and examined by MDCT and serum tumor markers CA199, CA242, and CEA, ③ patients were informed of the study and signed the consent form. Exclusion criteria were as follows: ① with signs of organ failure such as heart, lung, liver and kidney, ② without pathological diagnosis, ③ incomplete research data, ④ with other malignant tumors, ⑤ patients allergic to iohexol. There was no statistical difference in general data between the two groups (*p* > 0.05), with comparability. This study was approved by the ethics committee of our hospital (YYLUNLH20150230).

### MDCT examination

Discovery CT 750 HD CT (GE, USA) was used for MDCT examination. The patients were forbidden to eat or drink for 8 hours prior to the examination, which must be performed on an empty stomach. Before examination, patients drank 600 mL of water to fill the duodenum. All positions from the lower kidneys to the top of the diaphragm were sequentially scanned, followed by enhanced scans. The scanning parameters were 2.5 mm of interval and layer thickness, rotation time of 0.5 s/rot, field of view of 348 × 348, collimation of 0.5mm, scanning current and voltage of 300 mA and 120 kV respectively. The contrast agent iohexol was injected into the cubital vein at 3~4 mL/s. Arterial phase, parenchymal phase and delayed phase scanning were performed 20~25, 35~40, and 60~70 s after contrast injection, respectively. The images were processed on a MDCT system. The radiographic review was carried out by two imaging physicians, including the size, morphology, edge, degree of enhancement, nerve infiltration, lymph node metastasis, and tumor stage.

**Fig. 1:**
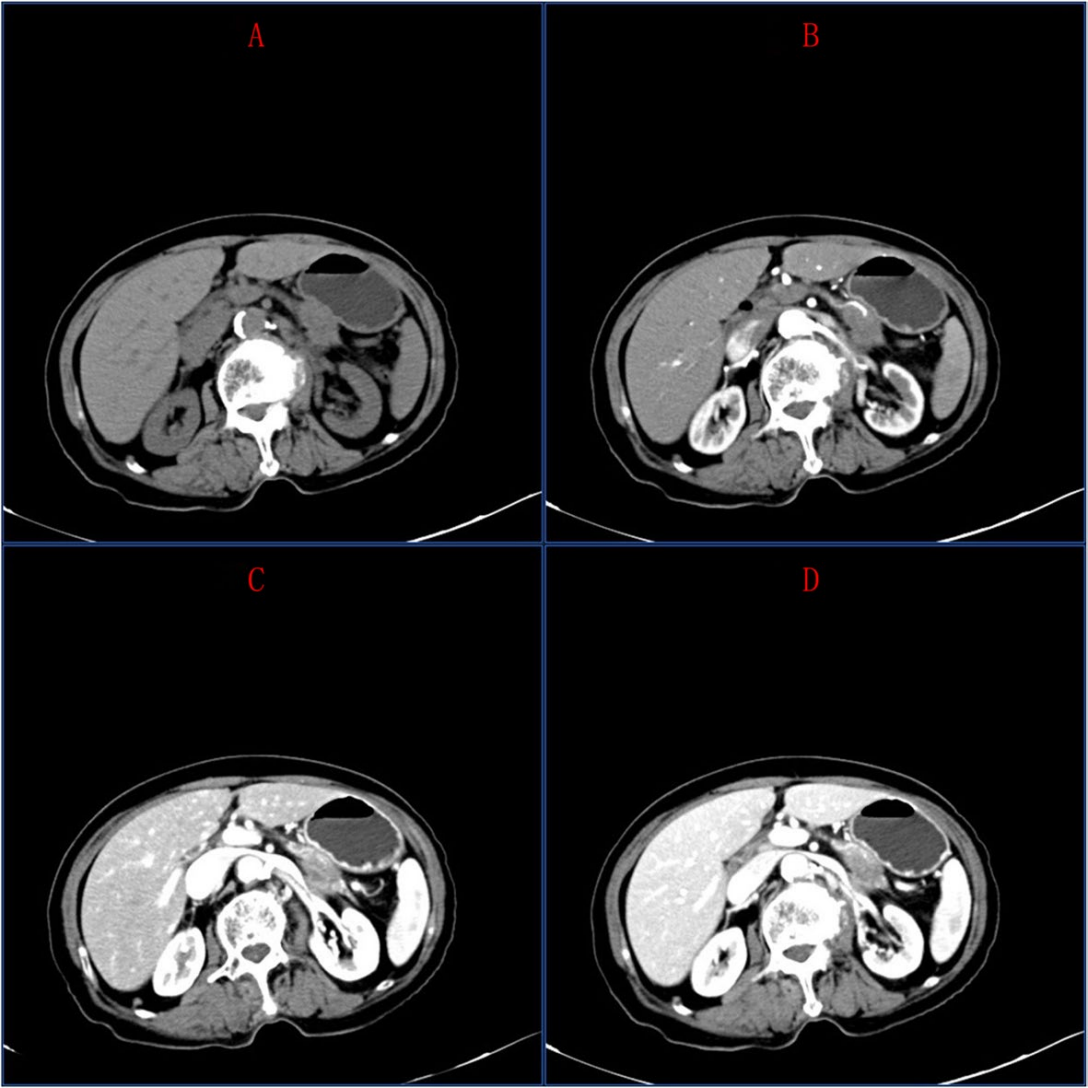
On plain scan, there was a low-density lesion in the tail of the pancreas. On enhancement examination, there was uneven enhancement, showing a relatively low-density lesion. The internal vessels of the pancreas were invaded and changed, including the adjacent left renal vein and the left adrenal gland

**Fig. 2:**
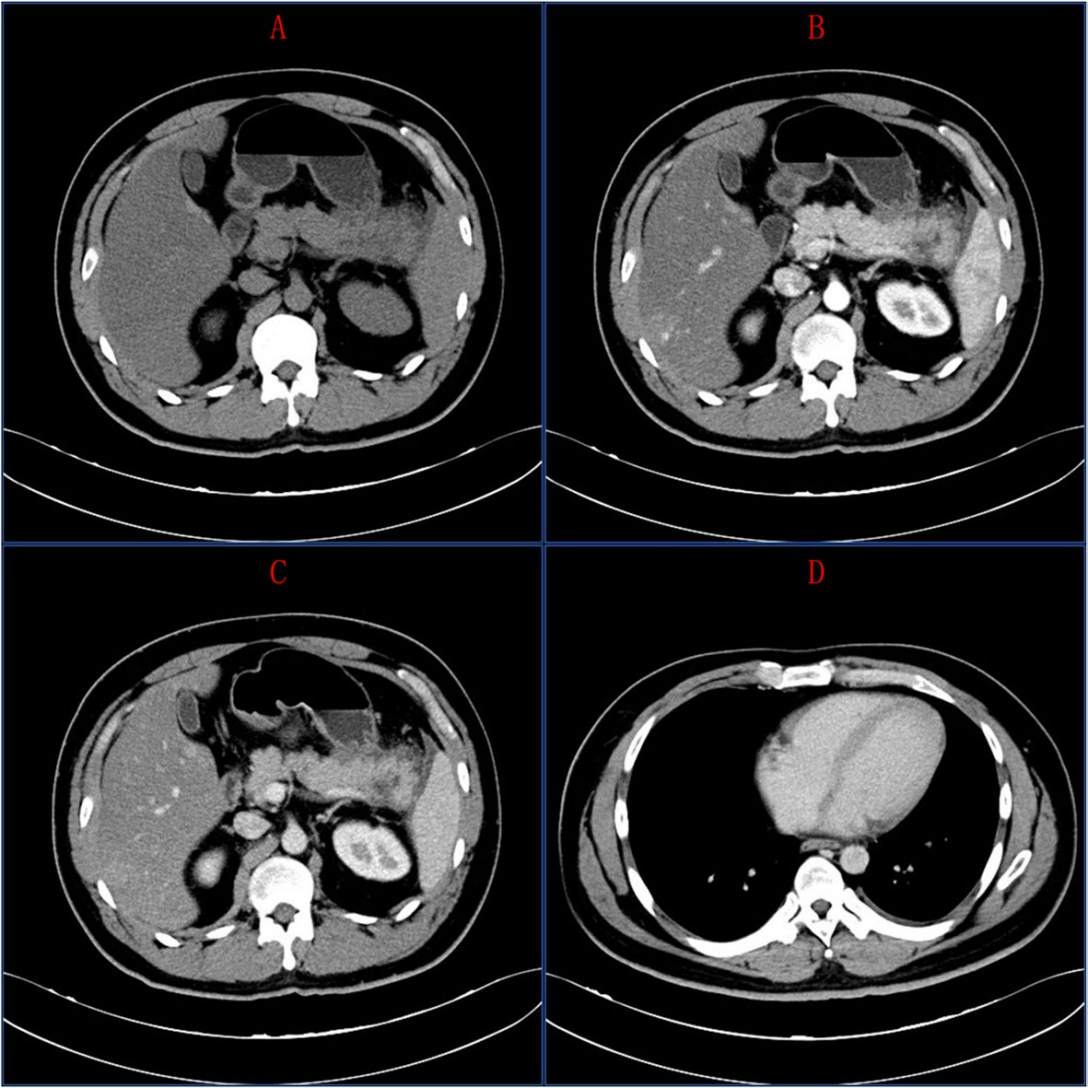
On plain scan, the pancreas tail was plump, uneven and slightly low-density shadow was observed locally, and the surrounding space was blurred with a little fluid shadow, but no obvious enhancement was observed. The left anterior renal fascia was thickened

MDCT of PC showed irregular spherical shape with unclear margin and maximum density difference from surrounding normal pancreatic parenchyma. MDCT scan images of PC are shown in Fig. 1. MDCT images of pancreatitis are shown in Fig. 2.

Preoperative clinical staging were as follows [9]: stage I: the diameter of the lesion was less than 2 cm, the lesion was confined to the pancreatic capsule without vascular involvement. Stage II: the tumor was 2–4 cm in diameter, and the capsule was infiltrated by cancer cells without vascular involvement. Stage III: the tumor diameter was over 4 cm, and lymph node metastasis was less than two stations, but with no distant metastasis. Stage IV: the tumor diameter was more than 4 cm, with lymph node metastasis at more than three stations and with distant metastasis.

### Serum tumor markers

3.5 ml elbow venous blood was extracted from the two groups on an empty stomach from 6:00–8:00 a.m., and the serum was separated by centrifugation after standing for self-coagulation. CA199, CA242, and CEA were detected by Electro-Chemiluminescence Immunoassay (Roche Elecsys-2010, Switzerland).

### Evaluation methodology

MDCT results of PC consistent with pathological diagnosis was defined as true positive, and inconsistent was defined as false negative MDCT results of benign pancreatic lesions consistent with the pathological diagnosis was defined as true negative, and inconsistent was false positive. Serum tumor marker less than or equal to the critical value was considered as negative, and greater than the critical value was judged as positive. Joint examination was judged as positive when one or more items were positive, and as negative when all items were negative.

### Follow-up

After treatment, patients came to the hospital for re-examination every 2 months, including imaging (CT, magnetic resonance, ultrasound) and serum tumor markers, plus 18F-FDG PET/CT if necessary. Follow-up was discontinued when recurrence, metastasis, or death occurred. No recurrence, metastasis or death occurred during the follow-up period, and the end point of the follow-up period was 24 months.

### Statistical analysis

SPSS19.0 statistical software was used for data analysis. Measurement data were expressed by mean ± standard deviation, and comparison between the two groups was performed by *t* test. The count data were expressed as rate (%), and *χ*^2^ test was used for comparison between groups. The diagnostic value of MDCT, CA199, CA242, and CEA in PC was calculated by four-grid table method. Kaplan-Meier method and log-rank were used to test survival analysis. *p* < 0.05 was considered statistically significant.

## Results

### Diagnostic analysis of PC and pancreatitis by MDCT

After MDCT examination of 85 PC, 69 cases (true positive) were consistent with pathological diagnosis, and 16 cases (false negative) were misdiagnosed. In 39 cases of pancreatitis, the results of MSCT were consistent with pathological diagnosis in 34 cases (true negative), and 5 cases were misdiagnosed as PC (false positive). The preoperative clinical staging of MDCT in 69 patients with PC was compared with postoperative pathological staging. The coincidence rate of stage I, II, III, and IV assessment was 70.00% (7/10), 62.96% (17/27), 72.72% (16/22), and 80.00 (8/10), respectively, and the overall coincidence rate was 69.57% (48/69) (Table 1).

**Table 1:** Evaluation of preoperative staging of PC by MDCT

MDCT	Surgical and pathological results			
	Stage I	Stage II	Stage III	Stage IV
Stage I	7	5	0	0
Stage II	3	17	4	0
Stage III	0	5	16	2
Stage IV	0	0	2	8
Total	10	27	22	10

### Comparison of serum CA199, CA242, and CEA expression levels between PC group and pancreatitis group

The levels of CA199, CA242, and CEA in patients with PC were sharply higher than those in patients with pancreatitis (*p* < 0.01, Table 2).

**Table 2:** Comparison of serum CA199, CA242, and CEA expression levels between the two groups

Group	Cases(*n*)	CA199 (U/mL)	CA242 (U/mL)	CEA(ng/mL)
PC group	85	325.63 ± 85.24	269.42 ± 77.36	165.34 ± 46.84
Control group	39	42.32 ± 10.29	46.74 ± 12.23	13.96 ± 2.45
*t*		20.642	17.45	20.136
*p*		< 0.01	< 0.01	< 0.01

### Correlation analysis of CA199, CA242, CEA expression levels, and clinicopathological factors in PC

The expression levels of CA199, CA242, and CEA in PC group had no significant correlation with gender, age, and tumor site (*p* > 0.05), but were notably correlated with clinical stage, differentiation degree, and distant metastasis (*p* < 0.01, Table 3). The levels of CA199, CA242, and CEA in patients with high stage, low differentiation, or distant metastasis were clearly higher than those in patients with low stage, high and middle differentiation, and no distant metastasis (*p* < 0.01, Table 3).

**Table 3:** Correlation analysis of CA199, CA242, CEA expression levels, and clinicopathological factors in PC group

	*n*	CA199 (U/mL)	CA242 (U/mL)	CEA(ng/mL)
Gender				
Male	56	328.24 ± 88.23	265.87 ± 78.23	168.71 ± 43.20
Female	29	320.59 ± 79.63	276.27 ± 80.02	158.83 ± 37.69
Age (years)				
< 60	37	318.88 ± 80.66	263.25 ± 68.24	163.22 ± 47.12
≥ 60	48	330.25 ± 92.24	274.17 ± 85.21	166.97 ± 49.20
Tumor site				
Head of pancreas	55	330.29 ± 89.36	273.54 ± 79.32	167.23 ± 45.23
Cauda pancreatitis	30	323.83 ± 79.25	261.89 ± 75.62	161.88 ± 50.72
Clinical stage				
I + II	53	165.98 ± 41.23	136.22 ± 36.78	96.41 ± 23.67
III + IV	32	590.05 ± 132.54^a^	470.03 ± 126.34^a^	279.50 ± 67.82^a^
Differentiation degree				
High and middle differentiation	34	156.84 ± 39.66	149.83 ± 41.85	112.06 ± 36.78
Low differentiation	51	428.16 ± 116.24^a^	448.80 ± 113.65^a^	218.08 ± 64.27^a^
Distant metastasis				
No	75	262.84 ± 62.30	217.37 ± 60.31	124.28 ± 32.64
Yes	10	796.55 ± 196.33^a^	659.74 ± 156.79^a^	423.52 ± 102.39^a^

^a^*p* < 0.01

### Comparison of MDCT combined with CA199, CA242, and CEA in diagnosis of PC

The results of MDCT, CA199, CA242, and CEA in diagnosis of PC were compared with the pathological diagnosis (Table 4). The comparison of single and combined examination of MDCT, CA199, CA242, and CEA in diagnosis of PC was shown in Table 5. The sensitivity, accuracy, and negative predictive value of MDCT combined with CA199, CA242, and CEA in the diagnosis of PC were sharply higher than those of each single examination (*p* < 0.01).

**Table 4:** The results of MDCT, CA199, CA242, and CEA in diagnosis of PC and pathological diagnosis

		Pathological diagnosis results	
		Malignant	Benign
MDCT	Malignant	69	5
	Benign	16	34
CA199	Malignant	61	6
	Benign	24	33
CA242	Malignant	58	4
	Benign	27	35
CEA	Malignant	55	5
	Benign	30	34
Combined detection	Malignant	82	7
	Benign	3	32

**Table 5:** Comparison of single and combined MDCT, CA199, CA242, and CEA in diagnosis of PC

Index	Sensitivity	Specificity	Accuracy	Positive predictive value	Negative predictive value
MDCT	81.18(69/85)	87.18(34/39)	83.06(103/124)	92.86(69/71)	68.18(34/50)
CA199	71.76 (61/85)	84.61(33/39)	75.81(94/124)	91.04(61/67)	57.89(33/57)
CA242	68.24(58/85)	89.74(35/39)	75.00(93/124)	93.55(58/62)	56.45(35/62)
CEA	64.71(55/85)	87.18()34/39)	71.77(89/124)	91.67(55/60)	53.13(34/64)
Combined detection	96.47(82/85)^a,b,c,d^	82.05(32/39)	91.94(114/124)^a,b,c,d^	92.13(82/89)	94.12(32/34)^a,b,c,d^
*χ*^2^	35.791	1.118	19.864	0.415	17.056
				0.981	

^a^*p* < 0.01, compared with MDCT group; ^b^*p* < 0.01, compared with CA199 group, ^c^*p* < 0.01, compared with CA242 group. ^d^*p* < 0.01, compared with CEA group

### Effects of CA199, CA242, and CEA levels on prognosis before treatment

**Fig. 3:**
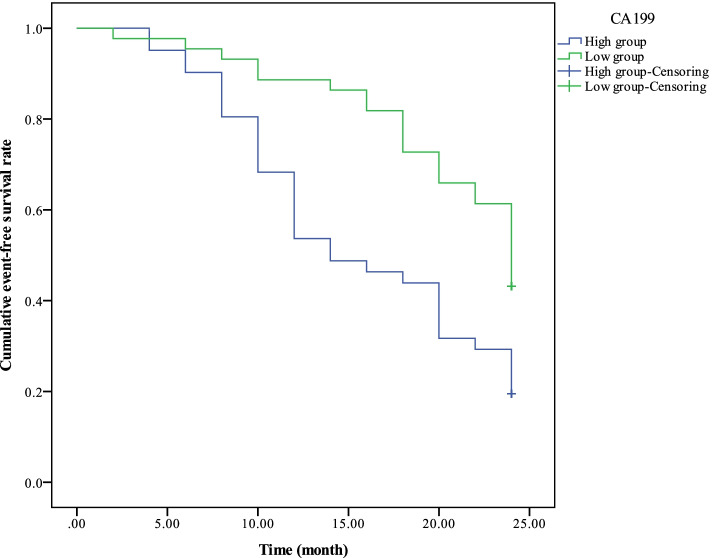
Comparison of 2-year event-free survival curve between the CA199 high expression group and the low expression group

**Fig. 4:**
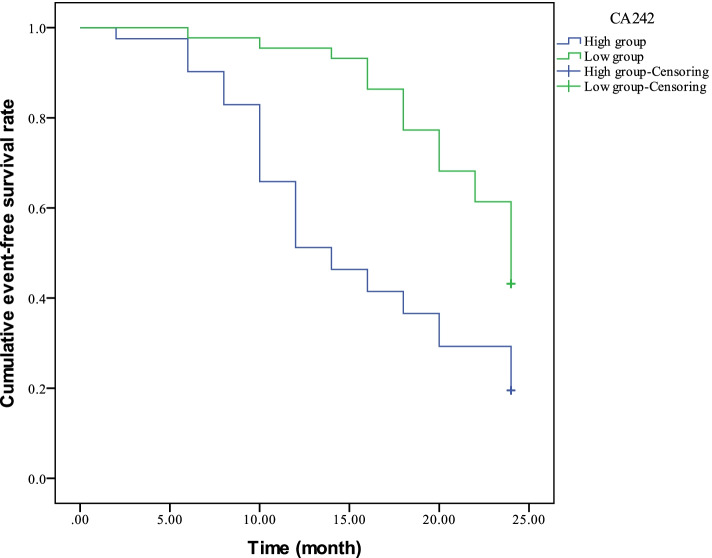
Comparison of 2-year event-free survival curve between the CA242 high expression group and the low expression group

**Fig. 5:**
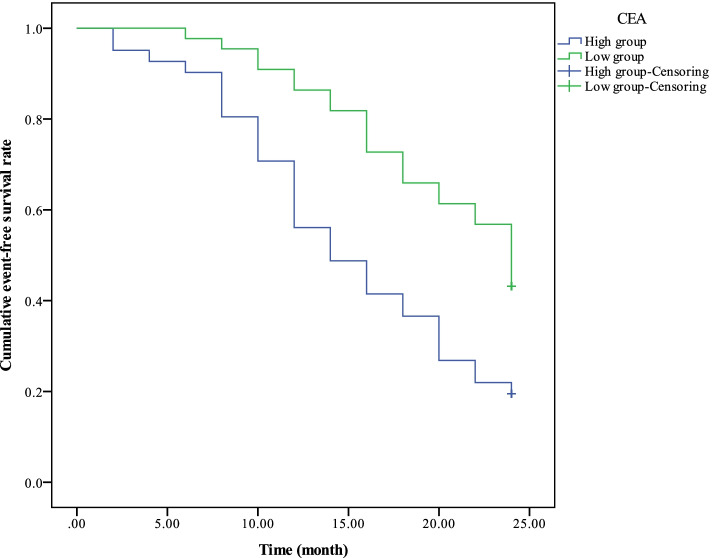
Comparison of 2-year event-free survival curve between the CEA high expression group and the low expression group

Before treatment, the 2-year event-free survival rate in high expression group of serum tumor markers (CA199 > 418.06 U/mL, CA242 > 389.46 U/mL, CEA > 203.44 ng/mL) was significantly lower than the low expression group (CA199 ≤ 418.06 U/mL, CA242 ≤ 389.46 U/mL, CEA ≤ 203.44 ng/mL) *(χ*^2^ = 9.746, 12.896, 10.212, *p* = 0.002, 0.000, 0.001, *p* < 0.01, Figs. 3, 4, and 5).

## Discussion

At present, MDCT has become the first choice for diagnosis, tumor staging and treatment planning of PC [10]. MDCT is a method to display the structure of tumor lesions by imaging the density difference between lesions and tissues [11]. MDCT in the diagnosis of PC can clearly observe the location, size, enhancement, adjacent fat space, and relationship with surrounding tissues, as well as pancreatic lymph node metastasis, vascular infiltration, and distant metastasis, which has certain advantages for preoperative evaluation of clinical stage [12]. All the patients in this study presented iso-density or low-density on MDCT plain scan, and the tumor density was similar to that of pancreatic parenchyma, so it was easy to miss diagnosis by plain scan. When dynamic enhanced scan was performed, the enhancement of the lesion was not obvious and showed a low-density shadow, while the surrounding pancreas was significantly enhanced and the density was relatively uniform, which made the outline and morphology of the tumor more clear [13]. The main reasons for misdiagnosis in this study may be that the lesions were small and easy to be missed. On the other hand, some tumors were difficult to be accurately located and may show atypical imaging manifestations. For example, in two cases of mass pancreatitis, the lesions were located in the head of the pancreas, and the lesions were compressed into the common bile duct and pancreatic duct, resulting in double duct sign. The other two patients had obvious low-density lesions on plain scan, and the enhancement pattern on enhanced scan was similar to that of PC, thus causing misdiagnosis. Preoperative staging of patients with PC can provide an important basis for making an accurate surgical plan. The results of this study showed that the overall coincidence rate between preoperative clinical staging and postoperative pathological staging of MDCT was 69.57%, which was basically consistent with the previous report [14]. Although MDCT is of great value in the diagnosis of PC, the sensitivity of the lesion to a diameter smaller than 2 cm causes a certain degree of misdiagnosis.

As a simple and non-invasive diagnostic method, serum tumor markers have been widely used in the diagnosis and prognosis evaluation of various tumors. A large number of studies have shown that biomarkers related to PC have certain guiding significance for early diagnosis and prognosis assessment, including CA199, CA242, CA50, CEA, etc. [15]. CA199 is the most widely used and effective tumor marker in the diagnosis of PC, and was once known as the ‘gold marker’ in the diagnosis of PC [16]. CA199, a Lewis blood group antigen, is significantly increased in the serum of PC patients [17]. However, CA199 was also increased in biliary tract obstruction, pancreatitis, and other digestive tract tumors, which limits its clinical application. Therefore, CA199 cannot be used as a separate indicator to distinguish PC from benign pancreatic diseases [18]. In addition, some people lack Lewis-a blood group antigen gene and do not express CA199. Even if PC occurs, they cannot synthesized CA199 resulting in false negative [19]. CA242 is mainly expressed in pancreatic and colon malignant tumors [20]. Serum CA242 level was increased in patients with PC, especially in patients with pancreatic head cancer [21]. CA242 expression was not affected by bile secretion and Lewis antigen [22]. Additionally, studies have shown [23] that CA242 was rarely expressed in patients with acute pancreatitis and biliary benign diseases, and basically not affected by acute pancreatitis and cholecystosis, so it can be used as a marker related to the diagnosis of PC. CEA, as a broad-spectrum tumor marker, is involved in cell adhesion and only exists in trace in serum of healthy persons [24]. CEA is widely used as a biomarker for colorectal cancer, but about 60% of patients with PC have elevated serum CEA level. Although the practical frequency is not as high as CA199, CEA can be used as an auxiliary diagnostic indicator for PC combined examination [25], especially in judging the recurrence and metastasis of PC [26]. Our study found that the levels of serum CA199, CA242, and CEA in PC group were clearly higher than those in control group. The levels of CA199, CA242, and CEA in PC group were not significantly correlated with age, sex, and tumor site, but were notably correlated with tumor size, differentiation degree, clinical stage, and metastasis. However, as an in vitro diagnostic test, the detection of tumor markers is prone to false positive and false negative due to the influence of internal and external factors, so it should be combined with imaging.

## Conclusion

To sum up, both imaging and serological examinations have their own advantages and disadvantages. MDCT combined with serum tumor markers in the diagnosis of PC can complement and confirm each other, significantly improve the sensitivity and accuracy compared with single examination, and have guiding significance for preoperative and prognostic evaluation.

## Data Availability

The datasets used and/or analyzed during the current study are available from the corresponding author on reasonable request.
